# Supplementary material for: HiCDiff: single-cell Hi-C data denoising with diffusion models
Source: Brief Bioinform. 2024 Jun 10;25(4):bbae279. doi: 10.1093/bib/bbae279 (PMC11163381; doi:10.1093/bib/bbae279)
Supplement: HiCDiff_support_final_bbae279 [file hicdiff_support_final_bbae279.docx]

**Supplemental Data for**

**HiCDiff: single-cell Hi-C data denoising with diffusion models**

| **Algorithm S1:** denoising a chromosomal contact matrix (x) in the unsupervised mode in the inference phase. ***Notations***: all the variables without bar (e.g., $y_{t} )$ denote the data (e.g., a chromosomal contact matrix) in the original space, and all the variables with bar (e.g.,$\bar{y}_{t}$) denote the data in the spectral space (the space of eigenvalues associated with a linear transformation matrix **H**). The variables with subscript $\theta$ connect the diffusion process between the original and spectral spaces. And a line started with **#** is a comment describing the following operations.  **Input:** a noisy chromosome contact matrix x, where the noise is controlled by the input noise level $\sigma_{x}$; $f_{\theta}$, a trained model |
| --- |
| 1: $y_{T}\mathcal{\sim N(}\boldsymbol{0,I})$, and $\mathbf{H}\sim$ the pre-defined linear degradation matrix – the identity matrix *I*. |
| 2: Do singular value decomposition (SVD) of **H**, $\mathbf{H}=\boldsymbol{U}\boldsymbol{\Sigma}\boldsymbol{V}^{\boldsymbol{⊺}}$, $\boldsymbol{\Sigma\sim}$ a rectangular diagonal matrix containing all singular values, e.g., $\boldsymbol{s}_{\boldsymbol{i}}$ in decreasing order**,** $\boldsymbol{\Sigma}^{\boldsymbol{\dagger}}\boldsymbol{\sim}$ the pseudo inverse of $\boldsymbol{\Sigma}$.  3: **#** Initialize the start variables at time T |
| For each i-th decreasing order singular value $s_{i}$  $\bar{x}^{(i)}$ $\sim$the $i$-th vector of $\bar{x}=\boldsymbol{\Sigma}^{\boldsymbol{\dagger}}\boldsymbol{U}^{\boldsymbol{⊺}}x$  $\bar{y}_{T}^{i} \sim$ the i-th vector of $\bar{y}_{T}=\boldsymbol{V}^{\boldsymbol{⊺}}\boldsymbol{y}_{\boldsymbol{T}}$  $y_{T}^{i} \sim$ the i-th vector of $\boldsymbol{y}_{\boldsymbol{T}}$ |
| $\bar{y}_{T}^{i}=\boldsymbol{V}^{\boldsymbol{⊺}}y_{T}^{i}$ if $s_{i}=0$, else $\bar{y}_{T}^{i}=\bar{x}^{(i)}+ \boldsymbol{V}^{\boldsymbol{⊺}}y_{T}^{i}$ $-\frac{\sigma_{x}}{s_{i}}$ |
| 4: **#** Perform the inverse inference process to recover $y_{0}$ from x iteratively  **For** $t= T, \ldots, 1$ **do** |
| 5: $Z=\mathcal{N(}\boldsymbol{0, I})$ if $t>1, else$ $Z=0$ |
| 6: **#** collect the predicted $y_{\theta, t-1}$ from pretrained model $f_{\theta}$in the original space as follows  $y_{\theta, t-1}=\frac{1}{\sqrt{a_{t}}}\left( y_{t}-\frac{\beta_{t}}{\sqrt{1-\bar{a}_{t}}}f_{\theta}\left( y_{t},t \right) \right)+\sqrt{1-\bar{a}_{t-1}}Z$, where $a_{t} :=1-\beta_{t}, \bar{a}_{t} :=\prod_{s=1}^{t} a_{s}$ |
| 7: **#** convert variables in the original space to the spectral space  $\bar{y}_{\theta, t-1}^{(i)}\sim$ $i$-th vector of $\bar{y}_{\theta, t-1}=\boldsymbol{V}^{⊺}y_{\theta,t-1}$ at time $t$, and $\bar{x}^{(i)}$ $\sim$the $i$-th vector of $\bar{x}=\boldsymbol{\Sigma}^{\boldsymbol{\dagger}}\boldsymbol{U}^{\boldsymbol{⊺}}x$ |
| 8: **#** collect the predicted $\bar{y}_{t-1}^{\left( i \right)}$ in the spectral space based on the i-th singular value $s_{i}$ for three different situations  For each i-th decreasing order singular value $s_{i}$  $\bar{y}_{t-1}^{\left( i \right)}=\bar{y}_{\theta, t-1}^{(i)}+\sqrt{1-\eta^{2}}\sigma_{t-1}\frac{\bar{y}_{t}^{\left( i \right)}-\bar{y}_{\theta,t-1}^{(i)}}{\sigma_{t}}+\eta\sigma_{t-1}$, if $s_{i}=0$  $\bar{y}_{t-1}^{\left( i \right)}=\bar{y}_{\theta, t-1}^{(i)}+\sqrt{1-\eta^{2}}\sigma_{t-1}\frac{\bar{x}^{(i)}-\bar{y}_{\theta,t-1}^{(i)}}{{\sigma_{x}}/{s_{i}}}+\eta\sigma_{t-1}$, if $\sigma_{t-1}<\frac{\sigma_{x}}{s_{i}}$  $\bar{y}_{t-1}^{\left( i \right)}=\left( 1-\eta_{b} \right)\bar{y}_{\theta,t-1}^{\left( i \right)}+\eta_{b}\bar{x}^{\left( i \right)}+\sigma_{t-1}-\frac{\sigma_{x}}{s_{i}}\eta_{b}$, if $\sigma_{t-1}\geq\frac{\sigma_{x}}{s_{i}}$ ,  where $\sigma_{t-1}=\sqrt{\frac{1-a_{t-1}}{a_{t-1}}}$, $\eta=1,$and $\eta_{b}=\frac{2\sigma_{t}^{2}}{\sigma_{t}^{2}+{\sigma_{x}^{2}}/{s_{i}^{2}}}$ |
| 9: **#** convert predicted $\bar{y}_{t-1}^{\left( i \right)}$ in the spectral space to the original space and concatenate all vectors to get the full matrix $y_{t-1}$  $y_{t-1}^{\left( i \right)}=\boldsymbol{V}\bar{y}_{t-1}^{\left( i \right)}$  For each i-th decreasing order singular value $s_{i}$  $y_{t-1}=concatenate(y_{t-1}^{\left( i \right)})$ |
| 10: **End For** |
| 11: **Return** $y_{0}$ as denoised data |

| **Algorithm S2:** denoising a chromosomal contact matrix (x) in the supervised mode in the inference phase.  **Input**: $x, a$ noisy chromosomal matrix; $f_{\theta}$, a trained model. |
| --- |
| 1: $y_{T}\mathcal{\sim N(}\boldsymbol{0,I})$ |
| 2: **For** $t= T, \ldots, 1$ do |
| 3: $Z=\mathcal{N(}\boldsymbol{0, I})$ if $t>1, else$ $Z=0$ |
| 4: $y_{t-1}=\frac{1}{\sqrt{a_{t}}}\left( y_{t}-\frac{\beta_{t}}{\sqrt{1-\bar{a}_{t}}}f_{\theta}\left( x, y_{t},t \right) \right)+\sqrt{1-\bar{a}_{t-1}}Z$, where $a_{t} :=1-\beta_{t}, \bar{a}_{t} :=\prod_{s=1}^{t} a_{s}$ |
| 5: **End For**  6: **Return** $y_{0}$ as denoised data |

| **Table S1**. The results of the four unsupervised and supervised HiCDiff and DDPM diffusion models as well as five other non-diffusion supervised deep learning methods for denoising one single-cell Hi-C data of *human cells 2 and 3 on human_cells_2_3_test_data* at two input noise levels (0.1 and 0.5). The results of the input data without denoising are also shown as the baseline. “*” and “**” denote the best and second-best results, respectively. The unsupervised HiCDiff1 and DDPM1 used the linear noise variance schedule in its forward process, while the supervised HiCDiff2 and DDPM2 used the sigmoid noise variance schedule. | | | | | | | | | | | | | | | | |
| --- | --- | --- | --- | --- | --- | --- | --- | --- | --- | --- | --- | --- | --- | --- | --- | --- |
| **Type** | **Model** | **Noise level = 0.1** | | | | | | **Noise level = 0.5** | | | | | | | | |
|  |  | **PSNR** | **SSIM** | **MSE** | | **SNR** | | **PSNR** | | **SSIM** | | **MSE** | | | **SNR** | |
| **Input Data** | --- | 28.9786 | 0.1868 | 0.0013 | | 30275 | | 15.0271 | | 0.0193 | | 0.0324 | | | 6097 | |
| **Unsupervised diffusion** | HiCDiff1 | **42.9328**** | **0.9699*** | **0.00005*** | | **144480**** | | **36.8585**** | | **0.9591**** | | **0.000225**** | | | **75193 **** | |
|  | DDPM1 | 42.7655 | 0.9680 | **0.00006**** | | 138340 | | 36.1697 | | 0.9533 | | 0.0003 | | | 67284 | |
| **Supervised diffusion** | HiCDiff2 | 42.0023 | 0.9698 | 0.000075 | | 118891 | | 35.4670 | | 0.9585 | | 0.00031 | | | 61838 | |
|  | DDPM2 | 41.5441 | 0.9598 | 0.000085 | | 112110 | | 35.3751 | | 0.9523 | | 0.00039 | | | 56137 | |
| **Supervised non-diffusion deep learning** | DeepHiC | 6.0564 | 0.0100 | 0.2391 | | 1992 | | 6.0547 | | 0.0092 | | 0.2392 | | | 2396 | |
|  | Loopenhance | 2.1454 | 0.0059 | 0.5885 | | 1582 | | 2.1472 | | 0.0058 | | 0.5883 | | | 1528 | |
|  | HiCPlus | 6.0483 | 0.0036 | 0.2438 | | 2903 | | 6.0398 | | 0.0035 | | 0.2514 | | | 2915 | |
|  | HiCSR | 25.9040 | 0.9298 | 0.0075 | | 16360 | | 25.8143 | | 0.9189 | | 0.0078 | | | 16289 | |
|  | ScHiCEDRN | **43.2613*** | **0.9697**** | **0.00005*** | | **147489*** | | **37.4017*** | | **0.9624*** | | **0.00019*** | | | **78468*** | |
|  | | | | | | | | | | | | | | | |  |
| **Table S2**. The results of the four unsupervised and supervised HiCDiff and DDPM diffusion models as well as five other supervised non-diffusion deep learning methods for denoising one individual single-cell Hi-C data of *drosophila cells 1 and 2 on drosophia_cells_test_data* at two input noise levels (0.1 and 0.5). The results of the input data without denoising are also shown as the baseline. “*” and “**” denote the best and second-best results, respectively. The unsupervised HiCDiff1 and DDPM1 used the linear noise variance schedule in its forward process, while the supervised HiCDiff2 and DDPM2 used the sigmoid noise variance schedule. | | | | | | | | | | | | | | | |  |
| **Type** | **Model** | **Noise level = 0.1** | | | | | | | **Noise level = 0.5** | | | | | | |  |
|  |  | **PSNR** | **SSIM** | | **MSE** | | **SNR** | | **PSNR** | | **SSIM** | | **MSE** | **SNR** | |  |
| **Input Data** | --- | 28.4453 | 0.2325 | | 0.0015 | | 91034 | | 14.5864 | | 0.0356 | | 0.0354 | 18482 | |  |
| **Unsupervised diffusion** | HiCDiff1 | **41.3910**** | **0.9729*** | | **0.00007*** | | **427813**** | | **34.2649*** | | **0.9482**** | | **0.0004*** | **180594**** | |  |
|  | DDPM1 | 41.0571 | 0.9606 | | 0.00018 | | 402197 | | 31.9191 | | 0.9343 | | 0.0007 | 137326 | |  |
| **Supervised diffusion** | HiCDiff2 | 38.9784 | **0.9656**** | | **0.00016**** | | 311240 | | 32.1794 | | 0.9426 | | **0.0006**** | 144410 | |  |
|  | DDPM2 | 37.2031 | 0.9554 | | 0.00025 | | 249838 | | 31.2399 | | 0.9344 | | 0.0010 | 125422 | |  |
| **Supervised non-diffusion deep learning** | DeepHiC | 6.6774 | 0.1249 | | 0.2092 | | 7517 | | 6.6766 | | 0.1238 | | 0.2092 | 7516 | |  |
|  | Loopenhance | 2.7553 | 0.1147 | | 0.5162 | | 4786 | | 2.7626 | | 0.1127 | | 0.3523 | 4790 | |  |
|  | HiCPlus | 6.6765 | 0.1231 | | 0.2093 | | 5554 | | 6.6494 | | 0.1218 | | 0.2043 | 7089 | |  |
|  | HiCSR | 14.9658 | 0.7941 | | 0.0397 | | 19306 | | 14.4831 | | 0.7793 | | 0.0448 | 18640 | |  |
|  | ScHiCEDRN | **41.6869*** | **0.9729*** | | **0.00007*** | | **433829*** | | **34.0881**** | | **0.9495*** | | **0.0004*** | **181713*** | |  |

| **Table S3**. The results of the four unsupervised and supervised HiCDiff and DDPM diffusion models as well as five other supervised non-diffusion deep learning methods for denoising population Hi-C data of *human_population_test_data data* at two input noise levels (0.1 and 0.5). The results of the input data without denoising are also shown as the baseline. “*” and “**” denote the best and second-best results, respectively. The unsupervised HiCDiff1 and DDPM1 used the linear noise variance schedule in its forward process, while the supervised HiCDiff2 and DDPM2 used the sigmoid noise variance schedule. | | | | | | | | | |
| --- | --- | --- | --- | --- | --- | --- | --- | --- | --- |
| **Type** | **Model** | **Noise level = 0.1** | | | | **Noise level = 0.5** | | | |
|  |  | **PSNR** | **SSIM** | **MSE** | **SNR** | **PSNR** | **SSIM** | **MSE** | **SNR** |
| **Input Data** | --- | 28.2283 | 0.8332 | 0.0015 | 1448099 | 14.5308 | 0.4198 | 0.0355 | 299486 |
| **Unsupervised diffusion** | HiCDiff1 | 27.6019 | 0.8967 | 0.0017 | 1348932 | **16.7427**** | **0.5031**** | **0.0215**** | **385109**** |
|  | DDPM1 | 26.1168 | 0.8804 | 0.0024 | 1131995 | 16.5844 | 0.4729 | 0.0223 | 378497 |
| **Supervised diffusion** | HiCDiff2 | **31.5966**** | **0.9766*** | **0.0008*** | **1911097*** | 16.5747 | 0.4954 | 0.0249 | 377742 |
|  | DDPM2 | 29.0613 | 0.9557 | 0.0012 | 1597274 | 15.3508 | 0.4384 | 0.0295 | 328521 |
| **Supervised non-diffusion deep learning** | DeepHiC | 7.7732 | 0.2839 | 0.1641 | 139409 | 7.6184 | 0.2070 | 0.1703 | 136823 |
|  | Loopenhance | 4.7532 | 0.2301 | 0.3299 | 98292 | 4.6358 | 0.1686 | 0.3391 | 96949 |
|  | HiCPlus | 7.7692 | 0.2744 | 0.1643 | 139339 | 6.8179 | 0.0659 | 0.2056 | 124487 |
|  | HiCSR | 30.8713 | 0.9697 | 0.0008* | 1901590 | 15.8383 | 0.4545 | 0.0263 | 347969 |
|  | ScHiCEDRN | **31.9009*** | **0.9748**** | **0.0005*** | **2038916*** | **16.9232*** | **0.5041*** | **0.0205*** | **393977*** |

| **Table S4**. The results of the four unsupervised and supervised HiCDiff and DDPM diffusion models as well as five other supervised non-diffusion deep learning methods for denoising population Hi-C data of *drosophia_population_test_data* at two input noise levels (0.1 and 0.5). The results of the input data without denoising are also shown as the baseline. “*” and “**” denote the best and second-best results, respectively. The unsupervised HiCDiff1 and DDPM1 used the linear noise variance schedule in its forward process, while the supervised HiCDiff2 and DDPM2 used the sigmoid noise variance schedule. | | | | | | | | | |
| --- | --- | --- | --- | --- | --- | --- | --- | --- | --- |
| **Type** | **Model** | **Noise level = 0.1** | | | | **Noise level = 0.5** | | | |
|  |  | **PSNR** | **SSIM** | **MSE** | **SNR** | **PSNR** | **SSIM** | **MSE** | **SNR** |
| **Input Data** | --- | 27.8107 | 0.5470 | 0.0017 | 245307 | 14.6267 | 0.0984 | 0.0348 | 54062 |
| **Unsupervised diffusion** | HiCDiff1 | **31.3634**** | **0.7598**** | **0.0008**** | **374926**** | **26.2581**** | **0.5909**** | **0.0033**** | **198208**** |
|  | DDPM1 | 30.3938 | 0.7058 | 0.0009 | 351776 | 25.2610 | 0.4070 | 0.0064 | 155862 |
| **Supervised diffusion** | HiCDiff2 | 28.9845 | 0.6319 | 0.0013 | 273396 | 23.3784 | 0.4617 | 0.0049 | 163345 |
|  | DDPM2 | 28.7563 | 0.6236 | 0.0014 | 266188 | 22.9597 | 0.4228 | 0.0055 | 156177 |
| **Supervised non-diffusion deep learning** | DeepHiC | 7.0799 | 0.1501 | 0.1915 | 23163 | 7.0685 | 0.1443 | 0.1921 | 23130 |
|  | Loopenhance | 4.2113 | 0.1171 | 0.3751 | 16530 | 4.3095 | 0.1017 | 0.3662 | 16731 |
|  | HiCPlus | 7.0788 | 0.1339 | 0.1916 | 23160 | 6.9511 | 0.1029 | 0.1977 | 22790 |
|  | HiCSR | 29.6669 | 0.4933 | 0.0012 | 294032 | 22.8838 | 0.3708 | 0.0054 | 137215 |
|  | ScHiCEDRN | **31.9009*** | **0.7689*** | **0.0007*** | **380340*** | **26.7303*** | **0.6110*** | **0.00239*** | **207566*** |

| **Table S5**. The results of the four unsupervised and supervised HiCDiff and DDPM diffusion models as well as five other supervised non-diffusion deep learning methods for denoising one individual single-cell Hi-C data of *drosophila cells 1 and 2 in drosophia_cells_test_data* at the input noise levels 0.1 across two different resolutions: 20 kb and 10 kb, respectively. The results of the input data without denoising are also shown as the baseline. “*” and “**” denote the best and second-best results, respectively. The unsupervised HiCDiff1 and DDPM1 used the linear noise variance schedule in its forward process, while the supervised HiCDiff2 and DDPM2 used the sigmoid noise variance schedule. | | | | | | | | | |
| --- | --- | --- | --- | --- | --- | --- | --- | --- | --- |
| **Type** | **Model** | **Resolution = 20 kb** | | | | **Resolution = 10 kb** | | | |
|  |  | **PSNR** | **SSIM** | **MSE** | **SNR** | **PSNR** | **SSIM** | **MSE** | **SNR** |
| **Input Data** | --- | 28.7729 | 0.1777 | 0.0014 | 101034 | 28.4241 | 0.2004 | 0.0014 | 131825 |
| **Unsupervised diffusion** | HiCDiff1 | **50.8707*** | **0.9937*** | **0.000010*** | **1246041*** | **52.0355*** | **0.9962*** | **0.000013*** | **2090963*** |
|  | DDPM1 | **50.1723**** | **0.9918**** | 0.000015 | **1071167**** | 42.9365 | **0.9954**** | 0.00004 | 1083572 |
| **Supervised diffusion** | HiCDiff2 | 49.6916 | 0.9904 | **0.000014**** | 1041123 | **50.0713**** | 0.9940 | **0.000018**** | **1640005**** |
|  | DDPM2 | 47.9123 | 0.9886 | 0.00002 | 850892 | 47.3175 | 0.9899 | 0.00003 | 1176171 |
| **Supervised non-diffusion deep learning** | DeepHiC | 6.2852 | 0.0509 | 0.2267 | 8158 | 6.8335 | 0.1366 | 0.2123 | 11601 |
|  | Loopenhance | 2.3854 | 0.0421 | 0.5561 | 5206 | 2.8163 | 0.1270 | 0.5256 | 6889 |
|  | HiCPlus | 6.2851 | 0.0508 | 0.2266 | 8157 | 6.7135 | 0.1364 | 0.2141 | 10791 |
|  | HiCSR | 18.4410 | 0.9251 | 0.02324 | 27114 | 14.3394 | 0.8395 | 0.0357 | 26408 |
|  | ScHiCEDRN | 48.7997 | 0.9878 | 0.00002 | 911069 | 47.2654 | 0.9915 | 0.000024 | 1171185 |

| **Table S6**. The results of the four unsupervised and supervised HiCDiff and DDPM diffusion models as well as five other supervised non-diffusion deep learning methods for denoising one individual single-cell Hi-C data of *drosophila cells 1 and 2 in drosophia_cells_test_data* at the input noise levels 0.1, with/without chromosomes 4, X and M included. The results of the input data without denoising are also shown as the baseline. “*” and “**” denote the best and second-best results, respectively. The unsupervised HiCDiff1 and DDPM1 used the linear noise variance schedule in its forward process, while the supervised HiCDiff2 and DDPM2 used the sigmoid noise variance schedule. | | | | | | | | | |
| --- | --- | --- | --- | --- | --- | --- | --- | --- | --- |
| **Type** | **Model** | **including chromosomes 4, X and M** | | | | **excluding chromosomes 4, X and M** | | | |
|  |  | **PSNR** | **SSIM** | **MSE** | **SNR** | **PSNR** | **SSIM** | **MSE** | **SNR** |
| **Input Data** | --- | 28.4453 | 0.2325 | 0.0015 | 91034 | 28.4729 | 0.2346 | 0.0014 | 73122 |
| **Unsupervised diffusion** | HiCDiff1 | **41.3910**** | **0.9729*** | **0.00007*** | **427813**** | **42.5150*** | **0.9747*** | **0.00006*** | **385682*** |
|  | DDPM1 | 41.0571 | 0.9606 | 0.00018 | 402197 | **41.7751**** | **0.9734**** | 0.00008 | **357554**** |
| **Supervised diffusion** | HiCDiff2 | 38.9784 | **0.9656**** | **0.00016**** | 311240 | 41.3530 | 0.9725 | **0.00007**** | 333851 |
|  | DDPM2 | 37.2031 | 0.9554 | 0.00025 | 249838 | 41.1696 | 0.9668 | 0.00008 | 326719 |
| **Supervised non-diffusion deep learning** | DeepHiC | 6.6774 | 0.1249 | 0.2092 | 7517 | 6.6390 | 0.1180 | 0.2228 | 5957 |
|  | Loopenhance | 2.7553 | 0.1147 | 0.5162 | 4786 | 2.7167 | 0.1083 | 0.5496 | 3793 |
|  | HiCPlus | 6.6765 | 0.1231 | 0.2093 | 5554 | 6.6381 | 0.1162 | 0.2228 | 5957 |
|  | HiCSR | 14.9658 | 0.7941 | 0.0397 | 19306 | 15.1818 | 0.8022 | 0.0261 | 16186 |
|  | ScHiCEDRN | **41.6869*** | **0.9729*** | **0.00007*** | **433829*** | **41.7390** | **0.9731** | **0.00007**** | 347707 |

| **Table S7**. The results of the four unsupervised and supervised HiCDiff and DDPM diffusion models as well as five other supervised non-diffusion deep learning methods for denoising the Hi-C data of *yeast* at the input noise levels 0.1. The results of the input data without denoising are also shown as the baseline. “*” and “**” denote the best and second-best results, respectively. The unsupervised HiCDiff1 and DDPM1 used the linear noise variance schedule in its forward process, while the supervised HiCDiff2 and DDPM2 used the sigmoid noise variance schedule. | | | | | |
| --- | --- | --- | --- | --- | --- |
| **Type** | **Model** | **Resolution = 10 kb** | | | |
|  |  | **PSNR** | **SSIM** | **MSE** | **SNR** |
| **Input Data** | --- | 26.8336 | 0.4913 | 0.0021 | 9096 |
| **Unsupervised diffusion** | HiCDiff1 | **33.0876**** | **0.9105*** | **0.00049**** | **18687**** |
|  | DDPM1 | 30.5879 | **0.9032**** | 0.00087 | 14013 |
| **Supervised diffusion** | HiCDiff2 | 32.8561 | 0.8836 | 0.00052 | 18195 |
|  | DDPM2 | 32.0592 | 0.8794 | 0.00062 | 16600 |
| **Supervised non-diffusion deep learning** | DeepHiC | 9.8572 | 0.5179 | 0.1033 | 1288 |
|  | Loopenhance | 5.7819 | 0.4809 | 0.2641 | 805 |
|  | HiCPlus | 9.8575 | 0.5170 | 0.1032 | 1289 |
|  | HiCSR | 8.5598 | 0.1958 | 0.1393 | 1109 |
|  | ScHiCEDRN | **33.6172*** | 0.8924 | **0.00043*** | **19862*** |

| **Table S8**. The results of the two supervised HiCDiff and DDPM diffusion models as well as five other supervised non-diffusion deep learning methods for denoising the single-cell Hi-C data of *drosophila cells 1 and 2 in drosophia_cells_test_data*, with asymmetric or symmetric noise added at the input noise levels 0.1. The results of the input data without denoising are also shown as the baseline. “*” and “**” denote the best and second-best results, respectively. The supervised HiCDiff2 and DDPM2 used the sigmoid noise variance schedule. | | | | | | | | | |
| --- | --- | --- | --- | --- | --- | --- | --- | --- | --- |
| **Type** | **Model** | **asymmetric noise** | | | | **symmetric noise** | | | |
|  |  | **PSNR** | **SSIM** | **MSE** | **SNR** | **PSNR** | **SSIM** | **MSE** | **SNR** |
| **Input Data** | --- | 28.4453 | 0.2325 | 0.0015 | 91034 | 30.0127 | 0.3245 | 0.0014 | 100183 |
| **Supervised diffusion** | HiCDiff2 | **38.9784**** | **0.9656**** | **0.00016**** | **311240**** | **40.7489**** | **0.9683**** | **0.00015**** | **293690**** |
|  | DDPM2 | 37.2031 | 0.9554 | 0.00025 | 249838 | 40.3573 | 0.9599 | 0.00028 | 282671 |
| **Supervised non-diffusion deep learning** | DeepHiC | 6.6774 | 0.1249 | 0.2092 | 7517 | 7.8321 | 0.1280 | 0.2008 | 8973 |
|  | Loopenhance | 2.7553 | 0.1147 | 0.5162 | 4786 | 3.0911 | 0.1209 | 0.4991 | 5603 |
|  | HiCPlus | 6.6765 | 0.1231 | 0.2093 | 5554 | 7.8989 | 0.1392 | 0.2010 | 8898 |
|  | HiCSR | 14.9658 | 0.7941 | 0.0397 | 19306 | 15.8929 | 0.8310 | 0.0293 | 22023 |
|  | ScHiCEDRN | **41.6869*** | **0.9729*** | **0.00007*** | **433829*** | **42.6389*** | **0.9805*** | **0.00007*** | **448695*** |

| **Table S9**. The impact of the variance schedule on the performance of HiCDiff on *human_population_test_data* at the input noise level of 0.1. | | | | | |
| --- | --- | --- | --- | --- | --- |
| **Type** | **Model** | **PSNR** | **SSIM** | **MSE** | **SNR** |
| Unsupervised, linear variance | HiCDiff1 | 27.6019 | 0.8967 | 0.0017 | 1348932.1 |
| Supervised, linear variance | HiCDiff2 | 29.6478 | 0.9625 | 0.0011 | 1708533.1 |
| Unsupervised, sigmoid variance | HiCDiff1 | 27.3687 | 0.9219 | 0.0018 | 1309345.1 |
| Supervised, sigmoid variance | HiCDiff2 | 31.5966 | 0.9766 | 0.0008 | 1911097.8 |


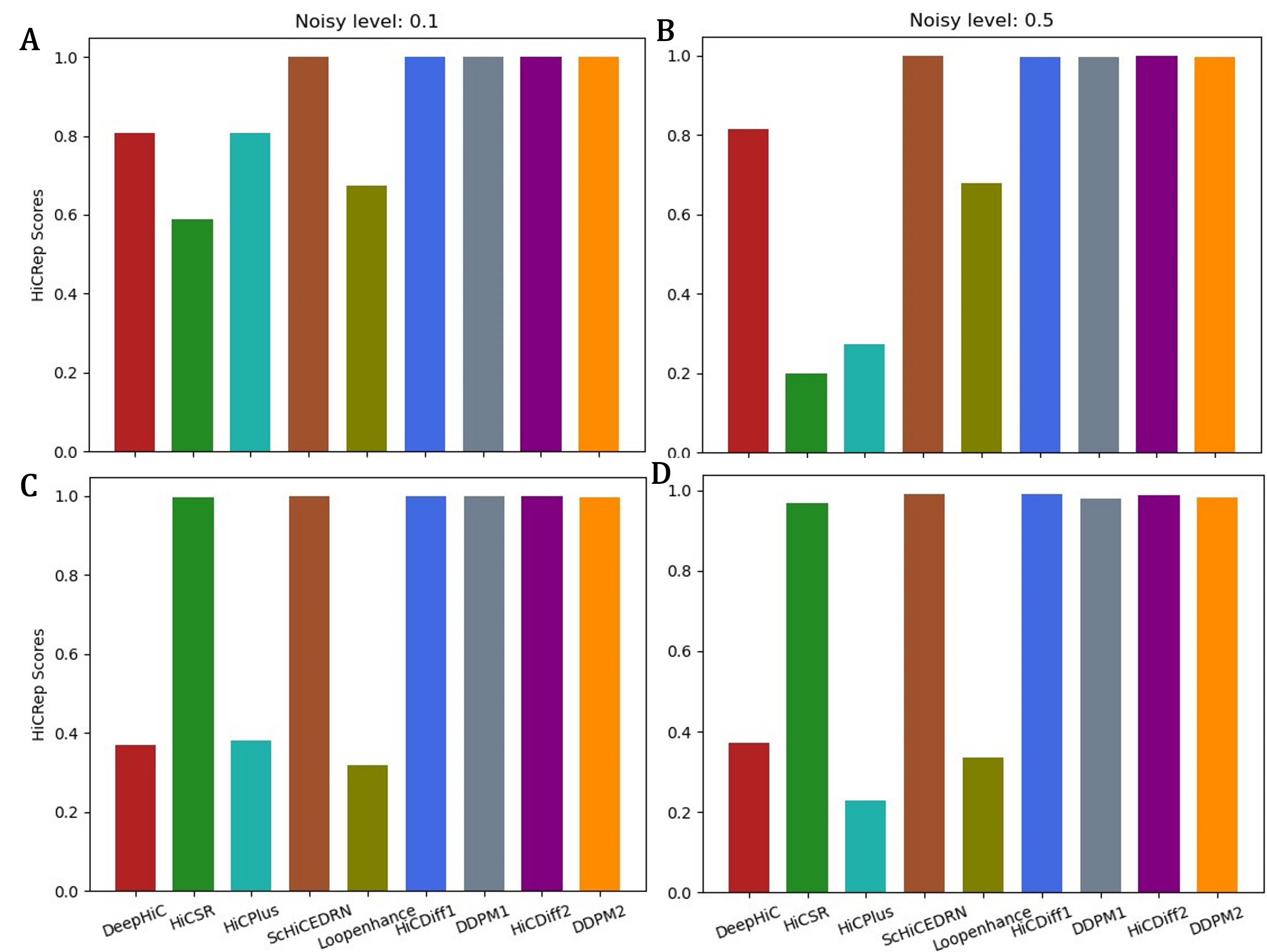


**Fig. S1.** The box plot of the average HiCRep scores from the *human_population_test_data* and *drosophila_population_test_data* Hi-C data at the different noise levels (0.1 and 0.5). (**A**) On human population cells at input noise level 0.1, (**B**) On human population cells at input noise level 0.5. (**C**) On Drosophila population cells at input noise level 0.1, and (**D**) On Drosophila population cells at input noise level 0.5.


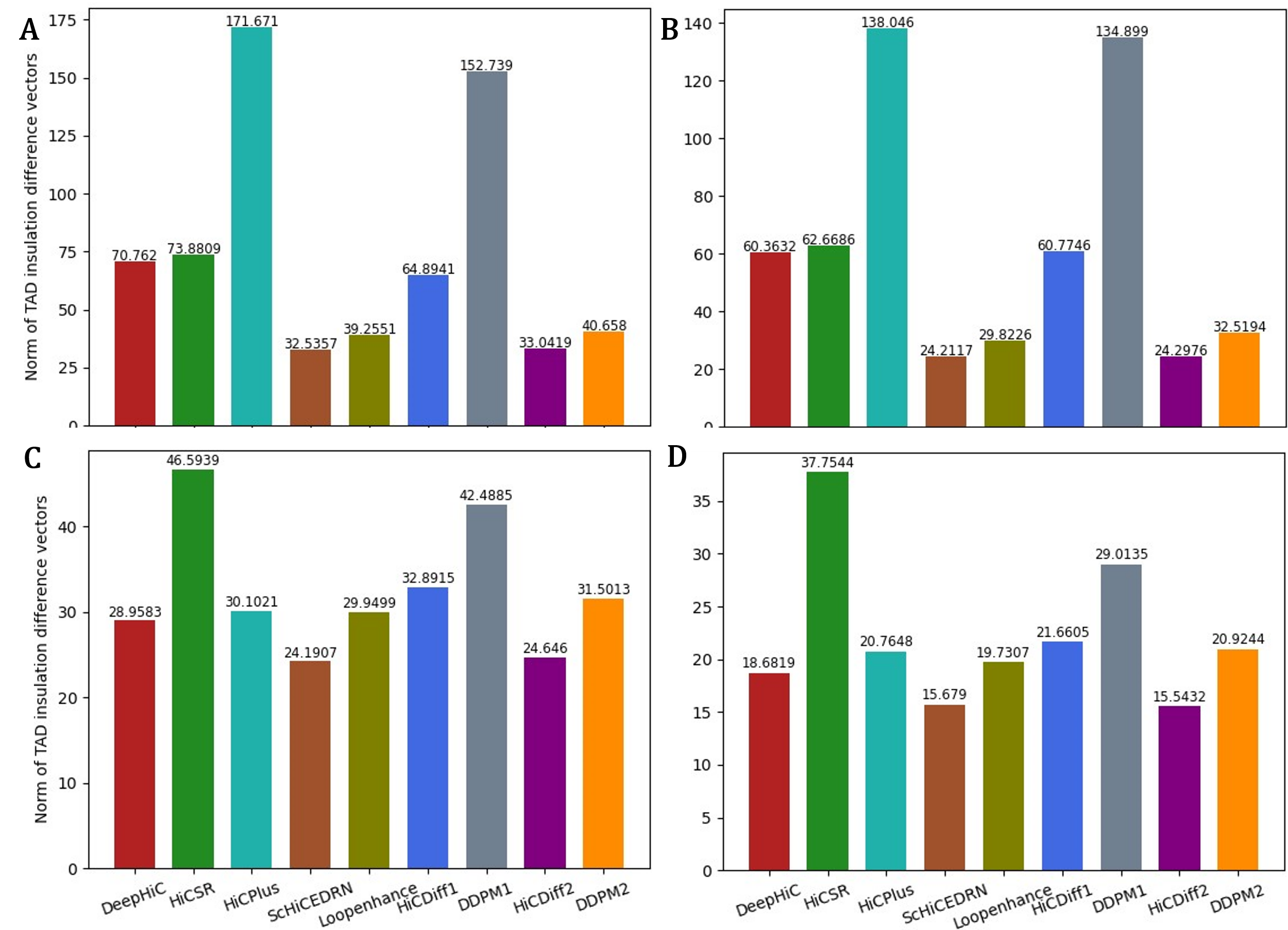


**Fig. S2.** L2 norm of the difference between the TAD insulation vectors computed from denoised matrices and the insulation vectors computed from the original high-resolution matrices across the cells and cell lines for the different methods. (A) Human cell 2, (B) Human cell 3, (C) Drosophila cell 1, and (D) Drosophila cell 2.
